# The assessment of sleep disturbances in cancer patients when measured with single items

**DOI:** 10.3389/fpsyg.2026.1812791

**Published:** 2026-09-14

**Authors:** Andreas Hinz, Jochen Ernst, Ute Goerling, Tanja Zimmermann, Beate Hornemann, Katja Petrowski, Anja Mehnert-Theuerkauf

**Affiliations:** 1 Department of Medical Psychology and Medical Sociology, Comprehensive Cancer Center Central Germany (CCCG), University Medical Center Leipzig, Leipzig, Germany; 2 Charité - Universitätsmedizin Berlin, Corporate Member of Freie Universität Berlin, Humboldt-Universität zu Berlin, and Berlin Institute of Health, Charité Comprehensive Cancer Center, Berlin, Germany; 3 Department of Psychosomatic Medicine and Psychotherapy, Hannover Medical School, Hannover, Germany; 4 Comprehensive Cancer Center, University Clinic Centre Dresden, Dresden, Germany; 5 Department of Medical Psychology and Medical Sociology, University Medical Center, Johannes Gutenberg University of Mainz, Mainz, Germany

**Keywords:** cancer survivorship, insomnia, quality of life, reliability, single-item measures, sleep disturbances, sleep quality

## Abstract

**Objective:**

Sleep disturbances are frequently observed in cancer patients. In addition to validated multi-item scales for assessing sleep disturbances, there are also single-item measures. The aim of this study was to investigate the extent to which such ultra-short single-item assessments of sleep disturbances provide useful information in cancer patients.

**Methods:**

This is a secondary analysis of a prospective multicenter study involving a sample of 970 patients with newly diagnosed cancer (mean age: 60.3 years; 46.8% women; mixed cancer diagnoses). We analyzed sleep disturbances using three single-item measures: the sleep items of the European-Organization-For-Research-And-Treatment-Of-Cancer Quality of Life Questionnaire (EORTC QLQ-C30) and the Patient Health Questionnaire-9 (PHQ-9), and the dichotomous sleep item included in the Problem List of the Distress Thermometer. Samples of the general population were used as comparison groups.

**Results:**

The sleep quality of the cancer patients was markedly poorer than that of the general population, with effect sizes between 0.67 and 0.74. The correlations between the sleep measures in the patients’ sample were between 0.61 and 0.74. The three sleep measures provided similar results regarding group differences based on sociodemographic and clinical variables, and their correlations with other scales of quality of life and mental health were also similar.

**Conclusion:**

Despite the lower reliability of the single-item measures of sleep quality in comparison with multi-item scales, these single items can help identify groups of patients who are at increased risk of sleep disturbances and the factors that contribute to poor sleep.

## Introduction

Sleep disturbances are frequently experienced by cancer patients (Büttner-Teleaga et al., 2021; Sun et al., 2023; Otte et al., 2015). While sleep disturbances are also common in the general population, their prevalence is markedly higher in patients and survivors of cancer (Hofmeister et al., 2022; Hinz et al., 2025). A meta-analysis of 160 individual studies identified an overall prevalence of sleep disturbances of 60.7% in cancer patients (Al Maqbali et al., 2022); however, there was considerable heterogeneity between the studies in terms of their reported prevalence rates. When comparing cancer patients with samples of the general population regarding multiple components of quality of life (QoL), fatigue and sleep disturbances were the variables displaying the strongest differences between these groups (Hinz et al., 2018).

Even though sleep disturbances are common and there are effective intervention methods for improving sleep quality (Høeg et al., 2025; Munir et al., 2025), oncologists tend to pay only insufficient attention to sleep disturbances experienced by their patients (Mercadante et al., 2017; Kwak et al., 2020). However, sleep disturbances in cancer patients are associated with low QoL (Liu et al., 2013), mental health problems (Price et al., 2023), and even mortality (Bach et al., 2021).

Gender and age play a role in determining the level of sleep disturbances of cancer patients. It is well-established that female cancer patients have poorer sleep quality than male patients (Hinz et al., 2025). While this gender-specific effect can also be observed in the general population, it is even more pronounced in cancer patients. For example, a summarizing study identified the gender effect in all eight samples of cancer patients it had analyzed (Hofmeister et al., 2022). With regard to age, several studies have found that the highest levels of sleep disturbances were experienced by younger patient group, whereas in the general population, older individuals were more likely to report sleep disturbances (Hofmeister et al., 2022).

A number of questionnaires have been developed for the reliable assessments of insomnia and sleep disturbances. These include the Pittsburgh Sleep Quality Index PSQI (Buysse et al., 1989) (19 items), the Insomnia Severity Index ISI (Morin et al., 2011) (7 items), the Athens Insomnia Scale (Soldatos et al., 2000) (8 items), and the Jenkins Sleep Scale JSS (Jenkins et al., 1988) (4 items). Several questionnaires on QoL or mental health also contain one item that is related to sleep disturbances, e.g., the EORTC QLQ-C30 (Aaronson et al., 1993), the PHQ-9 (Kroenke et al., 2001), and the GHQ-12 (Romppel et al., 2013). In addition, the Problem List of the Distress Thermometer (Mehnert et al., 2006b) contains a single yes-or-no item on sleep problems.

Healthcare providers and researchers in oncology who intend to assess QoL, symptoms, and specific features such as sleep disturbances, are interested in measurement tools that are reliable as well as economical. A larger number of items in a questionnaire increases the reliability of the measurement, yet also places a higher burden on patients who have to complete the questionnaire. Particularly in cases where patients are asked about a wide range of topics in addition to sleep disturbances, questionnaires often reach a length that may be stressful for patients. Therefore, it is relevant to investigate the extent to which single items on sleep disturbances that are already included in other questionnaires may suffice as assessment tools, avoiding the need for specific multi-item questionnaires. The analyses conducted on this topic to date are encouraging, yet not consistent. Comparisons between individual sleep-related items and multi-item sleep instruments yielded the following correlation coefficients: *r* (single sleep item, PSQI) = 0.44 (Delgado-Guay et al., 2011), *r* (GHQ-sleep item, JSS) = 0.52 (Lallukka et al., 2011), *r* (PHQ-9-sleep item, ISI) = 0.72 (Schulte et al., 2021), *r* (QLQ-C30 sleep item, JSS) = 0.73 (Hofmeister et al., 2020), *r* (QLQ C30-sleep item, PSQI) = 0.74 (Hinz et al., 2025), and *r* (single sleep item, JSS) = 0.79 (Klimt et al., 2023). The latter correlation coefficients are as high as those that describe the associations between established sleep instruments (Lin et al., 2020; Hinz et al., 2025).

When evaluating the usefulness of single-item measures for sleep disturbances, a number of criteria should be taken into consideration: the correlations between these sleep measurements, the correlations with related constructs of QoL, and known-groups comparisons. These groups can be patients vs. general population or, within the cohort of patients, groups defined by sociodemographic or clinical variables.

The majority of findings on the correlations between sleep scales and associations between sleep disturbances and sociodemographic and clinical variables have been obtained using multi-item sleep questionnaires. In this study, we aimed to evaluate whether such findings can also be obtained using single-item assessments.

The specific objectives of the present study were (a) to investigate the relationship between gender, age, and sleep disturbances, (b) to determine the associations between clinical factors and sleep disturbances, and (c) to analyze the associations between sleep disturbances, QoL, and mental health, based on single-item subjective measurements of sleep disturbances.

## Methods

### Sample of cancer patients

We analyzed secondary data from a prospective multi-center longitudinal observational study involving newly diagnosed cancer patients, which was conducted between April 2020 and July 2022. The study protocol and details of the study have previously been described (Mehnert-Theuerkauf et al., 2023; Goerling et al., 2024). Eligibility criteria included a diagnosis of a malignant solid tumor, age 18 or older, sufficient German language skills, and the physical, mental, and cognitive ability for participation. Exclusion criteria were a second malignancy or cancer relapse. All participants provided written informed consent prior to involvement in the study. Three German university comprehensive cancer centers (Leipzig, Berlin, and Hannover) as well as cooperating centers (Braunschweig, Dresden, and Göttingen) were included. We obtained ethics approval from the ethics committees of the participating centers (207/19-ek, SR-EK-536112020, 8533_BO_K_2019, 14/4/21Ü). The study was registered in the International Clinical Trials Registry (NCT04620564) (Mehnert-Theuerkauf et al., 2023).

In this present analysis, we restricted our analysis to the first measurement point up to 2 months after primary cancer diagnosis.

### Instruments

Medical data were obtained from the medical records of the participating study centers.

The test battery included several validated questionnaires. Among these, the EORTC QLQ-C30, the PHQ-9, and the Problem List (PL) of the Distress Thermometer contain one item each that refers to sleep disturbances.

### EORTC QLQ-C30

The EORTC QLQ-C30 (Aaronson et al., 1993) was designed to measure QoL in cancer patients. It comprises 30 items that can be assigned to five functioning scales, a global health status/QoL scale, three symptom scales, and six further single-item scales. One of these single-items scales refers to sleep disturbances: “Have you had trouble sleeping?” with four response options, coded from 1 to 4. This sleep item proved to be a valid measurement tool for sleep quality (Hofmeister et al., 2020). Each scale of the EORTC QLQ-C30, including the sleep scale, has to be transformed to the range 0–100. A sum score of the EORTC QLQ-C30 is defined as the aggregation of the functioning scales and the (inverted) symptom scales (Giesinger et al., 2016).

### PHQ-9

The Patient Health Questionnaire-9 PHQ-9 (Kroenke et al., 2001) was developed to measure depression with nine items. The four response options are coded with scores from 0 to 3. One of the items (item 3) refers to sleep disturbances: “trouble falling or staying asleep, or sleeping too much”. This item also proved to be valid for measuring sleep disturbances (Schulte et al., 2021).

### Distress thermometer (DT) and problem list (PL)

Distress was assessed using the German version of the DT (Mehnert et al., 2006b). It is a single-item visual analogue scale ranging from 0 (no distress) to 10 (extreme distress): Scores of 5 and above indicate a clinically significant level of distress (Donovan et al., 2014; Mehnert et al., 2006b). The Problem List (PL) contains 36 potential causes of distress (yes/no items, coded 1/0), one of the questions being the item “sleep problems” (Donovan et al., 2022).

### Gad-7

The seven-item Generalized Anxiety Disorder Screener GAD-7 (Löwe et al., 2008) was developed for measuring anxiety.

### FoP-Q-12

The Fear of Progression Questionnaire-12 (FoP-Q-12) (Mehnert et al., 2009; Mehnert et al., 2006a) is a 12-item short form of the 43-item Fear of Progression Questionnaire (FoP-Q) (Herschbach et al., 2005).

### SIS

We used the Stigma Impact Scale (SIS) (Fife and Wright, 2000) for measuring perceived stigmatization. The SIS comprises 24 items and four sub-scales. A high total score indicates a high level of perceived stigmatization.

### LS-3

The Loneliness Scale (LS-3) (Hughes et al., 2004) measures feelings of loneliness and social isolation with three items. A high sum score indicates a high level of loneliness.

### HLS-EU-Q16

Health literacy was measured using the European Health Literacy Survey Questionnaire (Gustafsdottir et al., 2020) with 16 items. High sum scores indicate high levels of health literacy.

### Karnofsky performance status scale

The Karnofsky Performance Status Scale (Schag et al., 1984) is an instrument for rating the ability of a patient to perform usual activities. The person is rated on a score ranging from 0 to 100. High scores indicate good performance.

### Statistical analysis

Statistics include descriptive parameters (mean and standard deviation). Group mean differences (between the cancer patients and the general population or between subgroups of the cancer patients) were expressed in terms of the effect size *d*, relating the mean score difference to the pooled standard deviation, and tested with t-tests for independent samples.

Associations between sleep variables and other continuous variables were expressed using Pearson correlations. To compare the sample of cancer patients with the general population, we used the data published in a normative study of the EORTC QLQ-C30 based on a sample representative of the German general population in terms of age and gender (*n* = 2,448) (Hinz et al., 2014). Since the distribution of age and gender in the patients’ sample differed from that of the normative study, we proceeded as follows to ensure a fair comparison. We built subgroups of the general population that matched the cancer group with regard to sex and age distribution. First, we calculated the percentages of sex and age groups in the cancer sample. Then we looked at the table of the normative scores and calculated weighted mean scores, using the percentages of the cancer sample as weighting factors. For example, the cancer sample included 137 men in the age range from 50 to 59 years (14.1% of the total sample). These percentages were used to calculate a general population mean score based on the published normative scores of the sleep item of the EORTC QLQ-C30. In this case, the general population mean score for men in the above age group was 13.2. This mean was weighted by 14.1%, resulting in an overall mean for the entire general population of 18.4. For the PHQ-9, we had access to the original data of the normative study (Kocalevent et al., 2013) and calculated the weighted scores accordingly. To our knowledge, there are no normative scores for the Problem List. Therefore, the comparisons with the general population are restricted to the sleep items of the EORTC QLQ-C30 and the PHQ-9. Associations between clinical variables and sleep variables were tested with three-way ANOVAs. The dependent variables were the sleep items, and the independent variables were the clinical variable of interest, and sex and age group as covariates. Effect sizes η^2^ were calculated to express the degree of explained variance. All statistics were performed with SPSS version 29.

## Results

### Characteristics of the sample

After an initial eligibility check based on medical records, a total of 3,327 cancer patients were found to be eligible. A more thorough eligibility assessment was then conducted by contacting these patients during their stay at the hospital over the phone. However, 1,625 patients could not be reached to confirm their eligibility. Of the remaining 1,702 eligible patients who could be reached, 1,150 patients agreed to participate. The latter were younger (60.4 vs. 65.9 years) with a better performance status (80.0 vs. 73.6) compared to patients who refused to participate. Further details of the comparison between participants and non-participants are provided elsewhere (Goerling et al., 2024). Of the 1,150 patients who declared that they were willing to participate, 1,003 completed the questionnaires (Goerling et al., 2024). We restricted our analyses to those participants who had no missing values in the three sleep-relevant instruments: the sleep item of the EORTC QLQ-C30 (C30-Sleep), the sleep item of the PHQ-9 (PHQ-9-Sleep), and the sleep item of the Problem List (PL-Sleep). This resulted in a final sample of 970 patients, 516 men (53.2%) and 454 women (46.8%). The mean age of the sample was 60.3 years (SD = 13.2 years; range: 21–92 years). Table 1 presents further characteristics of the sample.

**Table 1:** Characteristics of the sample (*N* = 970).

Characteristics	*n*	%
Age group		
18–49 years	175	18.0
50–59 years	258	26.6
60–69 years	284	29.3
≥70 years	253	26.1
Marital status		
Married	580	59.8
Single	154	15.9
Divorced/separated	95	9.8
Widowed	53	5.5
Missing	88	9.1
School education		
< 10 years	97	10.0
10–11 years	448	46.2
≥ 12 years	341	35.2
Missing	84	8.7
Employment status		
Employed	446	46.0
Unemployed	21	2.2
Retired	392	40.4
Other	22	2.3
Missing	89	9.2
Socioeconomic status		
Low	141	14.5
Medium	427	44.0
High	385	39.7
Missing	17	1.8
Karnofsky index		
10–60	135	13.9
70–80	221	22.8
90	195	20.1
100	230	23.7
Missing	189	19.5
Tumor site		
Prostate (C61)	183	18.9
Melanoma (C43-C44)	169	17.4
Digestive organs (C15-C26)	135	13.9
Breast (C50)	127	13.1
Female genital organs (C51-C68)	121	12.5
Head and neck (C00-C14)	76	7.8
Kidney/urinary tract (C64-C68)	68	7.0
Lung (C34)	32	3.3
Other	59	6.1
Tumor stage		
I	420	43.3
II	211	21.8
III	170	17.5
IV	125	12.9
Missing	44	4.5
Metastases		
No	812	83.7
Yes	116	12.0
Missing	42	4.3
Surgery		
No	154	15.9
Yes	816	84.1
Chemotherapy		
No	781	80.5
Yes	189	19.5
Radiation therapy		
No	802	82.7
Yes	168	17.3

### Sex and age differences, and comparisons with the general population

The mean scores for the sleep variables are presented in Table 2, stratified by sex and age group. Women reported markedly more sleep disturbances than men in all three variables, in both the clinical sample and the general population sample. With regard to age, sleep disturbances decreased with increasing age in our sample of patients, while an opposite trend was observed in the general population. This finding indicates that the difference between the patients’ group and the general population with regard to sleep disturbances was highest in the younger age ranges. While the effect sizes of this difference were 0.83 and 1.03 (for C30-Sleep and PHQ-9-Sleep, respectively) in the age group of 18–49 years, the oldest age group presented markedly lower effect sizes (0.42 and 0.41) (Table 2).

**Table 2:** Sleep disturbances by sex and age group.

Groups	QLQ-C30-Sleep					PHQ-9-Sleep					PL-Sleep
	Patients		Gen. Popul.			Patients		Gen. Popul.			Patients
	M	(SD)	M	(SD)	*d*	M	(SD)	M	(SD)	*d*	%^a^
Sex											
Men	32.7	(33.6)	15.5	(24.2)	0.60	1.15	(1.05)	0.55	(0.72)	0.68	44.0
Women	46.7	(38.3)	21.7	(27.2)	0.76	1.45	(1.04)	0.66	(0.75)	0.88	60.1
Age group											
18–49 years	41.9	(38.8)	15.2	(25.7)	0.83	1.36	(1.06)	0.47	(0.67)	1.03	60.0
50–59 years	43.5	(37.6)	16.9	(25.8)	0.84	1.35	(1.06)	0.56	(0.69)	0.90	57.7
60–69 years	37.7	(35.9)	19.3	(25.0)	0.60	1.32	(1.04)	0.66	(0.78)	0.73	47.9
≥70 years	34.8	(34.0)	22.2	(26.5)	0.42	1.15	(1.07)	0.77	(0.78)	0.41	43.5
Total	39.2	(36.5)	18.4	(25.6)	0.67	1.29	(1.06)	0.61	(0.74)	0.76	51.5

a Percentage of yes-answers; M, mean; SD, standard deviation; d, effect size. All mean score differences between the patients’ groups and the corresponding groups of the general population were statistically significant with *p* < 0.001.

### Associations between clinical variables and sleep quality

Table 3 presents mean scores of the sleep items, broken down by clinical cancer-related variables. In terms of the tumor sites, breast cancer patients and patients with female genital organs reported the highest levels of sleep disturbances, while prostate cancer patients experienced the least sleep disturbances. With regard to tumor stage, the most pronounced sleep disturbances were found for stages III (C30-Sleep) and IV (PHQ-9-Sleep and PL-Sleep). Metastases were slightly and non-significantly associated with sleep disturbances. Patients who had received surgery slept significantly better than those without surgery.

**Table 3:** Mean scores of sleep quality, broken down by clinical variables.

Clinical characteristics	QLQ-C30-Sleep		PHQ-9-Sleep		PL-Sleep
	M	(SD)	M	(SD)	% (yes)
Tumor					
Prostate	30.4	(31.3)	1.13	(1.03)	41.0
Skin	32.7	(35.5)	1.03	(1.03)	47.3
Gastrointestinal	44.9	(35.8)	1.52	(1.04)	56.3
Breast	50.4	(36.3)	1.53	(1.01)	62.2
Female genital organs	52.1	(41.9)	1.63	(1.08)	67.8
Head and neck	30.7	(32.6)	1.04	(0.93)	38.2
Urogenital organs	35.3	(34.5)	1.21	(1.09)	47.1
Lung	36.5	(34.2)	1.22	(1.01)	56.2
Others	39.0	(39.2)	1.31	(1.09)	49.1
*F; p;* η^2^	*F* = 2.62; η^2^ = 0.018	***p* = 0.008**	*F* = 3.45; η^2^ = 0.021	***p* < 0.001**	*F* = 1.67; *p* = 0.102; η^2^ = 0.010
Stage					
I	37.5	(36.4)	1.18	(1.02)	51.9
II	40.0	(36.9)	1.39	(1.06)	51.2
III	42.7	(37.4)	1.38	(1.10)	53.5
IV	40.3	(36.0)	1.42	(1.07)	50.4
*F; p;* η^2^	*F* = 1.76; η^2^ = 0.005	*p* = 0.154	*F* = 4.20; η^2^ = 0.014	***p* = 0.006**	*F* = 0.25; *p* = 0.862; η^2^ = 0.001
Metastases					
No	39.0	(36.6)	1.27	(1.05)	51.6
Yes	41.7	(37.3)	1.47	(1.06)	54.3
*F; p;* η^2^	*F* = 0.47; η^2^ = 0.000	*p* = 0.494	*F* = 3.28; η^2^ = 0.003	*p* = 0.070	*F* = 0.06; *p* = 0.807; η^2^ = 0.000
Karnofsky index					
10–60	46.4	(38.2)	1.42	(1.02)	56.3
70–80	44.9	(37.3)	1.48	(1.07)	60.2
90	39.7	(35.8)	1.29	(1.02)	48.7
100	32.9	(34.8)	1.13	(1.07)	47.4
*F; p;* η^2^	*F* = 4.00; η^2^ = 0.016	***p* = 0.008**	*F* = 3.56; η^2^ = 0.015	***p* = 0.014**	*F* = 2.17; *p* = 0.091; η^2^ = 0.008
Surgery					
No	45.7	(36.1)	1.56	(1.10)	58.4
Yes	38.0	(36.5)	1.24	(1.04)	50.2
*F; p;* η^2^	*F* = 5.54; η^2^ = 0*.006*	***p* = 0.019**	*F* = 12.27; η^2^ = 0*.013*	***p* < 0.001**	*F* = 2.94; *p* = 0.087; η^2^ = 0*.003*
Chemotherapy					
No	38.8	(36.6)	1.27	(1.06)	51.2
Yes	40.9	(36.2)	1.39	(1.05)	52.9
*F; p;* η^2^	F = 0.016; η^2^ = 0*.000*	*p* = 0.898	F = 0.62; η^2^ = 0*.001*	*p* = 0.431	*F* = 0.369; *p* = 0.544; η^2^ = 0*.000*
Radiation therapy					
No	38.9	(36.4)	1.27	(1.04)	50.7
Yes	41.1	(37.2)	1.41	(1.11)	55.4
*F; p;* η^2^	*F* = 0.21; η^2^ = 0*.000*	*p* = 0.649	F = 2.14; η^2^ = 0*.002*	*p* = 0.144	*F* = 0.70; *p* = 0.403; η^2^ = 0*.001*

Bold: statistically significant.

### Correlations between the sleep scales and other scales

The correlations between the three assessments of sleep disturbances were as follows: *r* (C30-Sleep, PHQ-9-Sleep) = 0.74, 95% CI: [0.71–0.77]; *r* (C30-Sleep, PL-Sleep) = 0.62, 95% CI: [0.58–0.66], and *r* (PHQ-9-Sleep, PL-Sleep) = 0.61, 95% CI: [0.57–0.65]. Table 4 shows the correlations between the sleep measures and their correlations with other variables. Both the PHQ-9 score and the QLQ-C30 total score include one sleep item each, which artificially increases the correlation between the total score and that sleep item. Therefore, total scores for the QLQ-C30 and the PHQ-9 were also calculated by excluding the corresponding sleep item.

**Table 4:** Correlations between sleep items and other variables.

Scales	QLQ-C30-Sleep	PHQ-9-Sleep	PL-Sleep
EORTC QLQ-C30			
Functioning scales			
Physical	−0.37	−0.33	−0.26
Role	−0.33	−0.27	−0.20
Emotional	−0.47	−0.41	−0.35
Cognitive	−0.40	−0.34	−0.28
Social	−0.37	−0.35	−0.27
Global QoL	−0.40	−0.40	−0.30
Symptom scales			
Fatigue	0.53	0.45	0.35
Nausea/Vomiting	0.32	0.28	0.24
Pain	0.38	0.31	0.29
Dyspnea	0.28	0.22	0.19
Appetite loss	0.37	0.32	0.26
Constipation	0.28	0.26	0.19
Diarrhea	0.23	0.19	0.19
Financial difficulties	0.23	0.20	0.18
Sum score	−0.65	−0.54	−0.45
Sum score except sleep item	−0.56	−0.47	−0.38
Mental health			
PHQ-9 (Depression)	0.57	0.67	0.48
PHQ-9 except sleep item	0.48	0.52	0.40
GAD-7 (Anxiety)	0.43	0.43	0.37
FoP (Fear of progression)	0.37	0.35	0.32
HLS (Health literacy)	−0.14	−0.14	−0.14
SIS (Stigmatization)	0.18	0.18	0.14
LS-3 (Loneliness)	0.21	0.24	0.21

All correlation coefficients are statistically significant at *p* < 0.001.

Among the scales listed in Table 4, the EORTC QLQ-C30 sum score and depression (PHQ-9) were most strongly associated with sleep disturbances. Even after removing the sleep items form these scales, the correlations remain highest. Regarding the scales of the EORTC QLQ-C30, the correlations with the QLQ-C30 sleep item were somewhat higher than those with the PHQ-9 sleep item, while the weakest correlations were found for the sleep item of the Problem List. The rank order of the correlations, however, is very similar for the three sleep measurement tools, with high coefficients for the EORTC QLQ-C30 sum score and depression, and lowest correlations with health literacy and stigmatization. Among the functioning scales of the EORTC QLQ-C30, emotional functioning was most strongly associated with sleep for all three assessment tools.

## Discussion

The study confirmed severe sleep disturbances in cancer patients. Though sleep disturbances are also common in the general population, the strong effect sizes (*d* = 0.78 for QLQ-C30-Sleep and *d* = 0.73 for PHQ-9-Sleep) demonstrate that the sleep quality of the patients is markedly poorer. This result is in line with other studies that compared cancer patients with samples of the general population. A large oncological study with mixed cancer diagnoses that used the PSQI to assess sleep quality found an effect size of *d* = 0.90 when comparing cancer patients and the general population (Hinz et al., 2025). Another study using several sleep-related questionnaires with cancer patients (Friedrich et al., 2024) expressed the difference between cancer patients and the general population in terms of T scores (Mean = 50; SD = 10) and found that patients with an average level of sleep disturbances among cancer patients (T = 50) had a T score of about 59 in the context of the general population, i.e., nearly one standard deviation above the mean of the general population, corresponding to an effect size of *d* = 0.90. Our study showed that this difference in sleep disturbances between cancer patients and the general population can also be detected with single-item measures.

It is well-known that women generally report poorer sleep than men, which has been evidenced by several normative studies (Hinz et al., 2017; Tibubos et al., 2020; Zeitlhofer et al., 2000). Various factors that may explain this gender effect are discussed: hormonal factors (La et al., 2020), differences in the circadian rhythm (Duffy et al., 2011), and differences in stress responses and maladaptive cognitive patterns like rumination (Kim and Suh, 2019). Experiencing a cancer diagnosis does not reduce this gender gap; the difference between female cancer patients and women from the general population was even higher than that between male patients and men of the general population. This means that female cancer patients deserve special attention regarding their sleep disturbances.

In terms of age, all three sleep measures showed a decline of sleep disturbances with increasing age. Since this trend is not observed in the general population, the study shows that sleep disturbances particularly affect younger patients. Possible reasons for increased psychological distress and sleep disturbances among younger cancer patients include: disruptions to their overall life plans, difficulties in fulfilling their roles in partnership and family, concerns about their children, and problems with their ability to work (Jäkel et al., 2023; Fortmann et al., 2021). For younger people in particular, professional work is a source of financial stability and an important means of social participation (Brock et al., 2026). While the findings regarding age- and gender-related variations in sleep quality among cancer patients are not novel, our study demonstrates that these results can also be obtained by using single-item measurements.

With regard to the associations between clinical variables and sleep quality, in most cases the three measures yielded similar results: with the highest levels of sleep disturbances associated with female genital cancer, stages III or IV, low Karnofsky index, and not having undergone surgery. It should first be noted that clinical variables such as tumor type and gender may be confounding factors, so it remains unclear at this point to what extent the relatively high incidence of sleep disturbances among breast cancer patients can be attributed to gender. The ANOVA results given in Table 3 are adjusted for gender and age, and a significant main effect of a clinical factor means that this factor has an effect independent of gender and age. However, the focus of this study was on the similarity and dissimilarity of the results obtained with different sleep measurement tools. The significance levels differ between the three sleep measurement methods, with the lowest significance found for the associations of the Problem List. Generally speaking, QLQ-C30-Sleep and PHQ-9-Sleep showed similar results, even with regard to statistical significance, with a significant effect of tumor type, Karnofsky index and surgery, and non-significant associations with metastases, chemotherapy and radiation therapy. Here we want to note that our aim is not to discuss possible reasons for the lower sleep quality of patients showing a low Karnofsky index or those without surgery. Instead, our focus is on the similarity in assessment of the different tools measuring sleep quality.

The correlations between the two sleep items with four response options each, QLQ-C30-Sleep and PHQ-9-Sleep (*r* = 0.74) was slightly higher than the correlations between these two items and the dichotomous item of the Problem List (*r* = 0.61 and *r* = 0.62, respectively). Correlations between multi-item-sleep scales generally reach slightly higher coefficients: *r* (PSQI, ISI) = 0.84 (Cho et al., 2014); *r* (PSQI, ISI) = 0.79 (Dieck et al., 2018), *r* (PSQI, ISI) = 0.58 (Lin et al., 2020); *r* (PSQI, ISI) = 0.81 (Hinz et al., 2025), *r* (PSQI, JSS) = 0.72 (Hinz et al., 2025), *r* (Athens Insomnia Scale, ISI) = 0.64 (Lin et al., 2020), and correlations between single-item sleep scales and multi-item sleep scales were in between (e.g., *r* (PSQI, QLQ-C30-Sleep) = 0.74) (Hinz et al., 2025). The correlation between the QLQ-C30 sleep item and the PHQ-9 sleep item obtained in our study (*r* = 0.74) indicates that these two single-item multi-level measurements are, in contrast to the single-item dichotomous measurement of the Problem List (*r* = 0.61 and *r* = 0.62), suitable for assessing sleep quality with a sufficient degree of precision.

The correlations between the sleep items and other variables of QoL were highest for the QLQ-C30 sleep item. This may be due to the fact that the QLQ-C30 sleep item and the other scales of the EORTC QLQ-C30 share the same answer format with four identical response options. Regarding the other scales of mental health, the correlations of the QLQ-C30 sleep item were nearly identical to those of the PHQ-9 sleep item. Concerning the high association between PHQ-9-Sleep and the PHQ-9 sum score (*r* = 0.67), one hast to take into account that the sleep item of the PHQ-9 is already part of this questionnaire, which inflates this correlation. However, this correlation remains relatively high even after excluding the sleep item from the PHQ-9 total score (*r* = 0.52).

The correlations of the sleep item of the Problem List were lower than those of the other questionnaires. This may be explained by the more limited (two) response options of this measure. However, it should be noted that the sequence of the correlations in Table 4 is very similar for the three sleep measures. If one is interested in investigating whether sleep disturbances are more strongly correlated with depression than with anxiety or more strongly correlated with emotional functioning in comparison with physical functioning, all three measures provide the same answer. One can thus assume that the use of an established multi-item instrument for measuring sleep quality would lead to the same order, though on a slightly higher level because of the higher overall reliability.

We examined the similarity and dissimilarity of the three sleep measures with respect to several criteria: differences between the cancer patients and the general population, correlations between the sleep measures, associations with clinical variables and sleep, and associations with other scales of QoL and mental health. The general finding is that the three measures provided similar results, with a stronger similarity between the measures with four response options in comparison with the dichotomous item of the Problem List. This is an argument for using such single-item measures when research questions on a group level have to be answered. For such an application, we can recommend the use of the sleep item of the EORTC QLQ-C30 or the PHQ-9. However, there are no generally accepted cutoffs for the sleep items of the EORTC QLQ-C30 and the PHQ-9, and our study does not allow us to derive such cutoff values either. This limits the applicability of these single-item measures in clinical practice. The question arises in what contexts it would be appropriate to use one of the two sleep items. In general, it is not recommended to extract individual items from a questionnaire and present only those items without using the complete instrument. However, if patients’ quality of life or depressive symptoms are being assessed and the EORTC QLQ-C30 or PHQ-9 are therefore being used, it appears justified to consider the sleep item of the respective questionnaire instead of applying a multi-item tool to assess sleep quality.

The three sleep quality assessment tools belong to the category of subjective measurement. Over the last decades, objective measurement via polysomnography and actigraphy has gained increasing importance (Barsasella et al., 2021; Budhrani et al., 2015). However, the associations between subjective and corresponding objective measurements are generally relatively low (Kreutz et al., 2021; Palesh et al., 2014; Jakobsen et al., 2020). We did not include objective measurements, and, therefore, have to underline that all results obtained in this study refer to the subjective assessment of sleep.

At least in part, the correlations between the (subjective) sleep assessments can be traced back to a certain response set or acquiescence effect (Hinz et al., 2007). The use of objective measures could probably help clarify to which degree the correlations can be explained by such response style effect. However, the use of objective methods is costly, and the aim of this study was to examine how meaningful statements about sleep quality can be made with minimal effort.

Some limitations of this study should be mentioned. The aim of the study was to test ultra-short, single-item measures for sleep quality. It is self-evident that these single items are not as reliable as multi-item instruments, and, therefore, the correlations with other variables of QoL and mental health as well as sex and age differences and the differences regarding clinical categories are not entirely reliable. However, if differences nevertheless become statistically significant, it is probable that the actual differences (as assessed with more reliable instruments) would be even somewhat stronger. We did not include a validated instrument as a “gold standard” in the study, as there is no such “gold standard” for assessing sleep disturbances, and the correlations between established instruments for measuring sleep quality are often only around 0.80 (Hinz et al., 2025). A further limitation is that, as already discussed above, we cannot relate the sleep assessments of this study to objective sleep parameters. Moreover, this study is a cross-sectional study; consequently, it cannot capture dynamic changes in sleep quality. The data were also collected at a German rehabilitation clinic; therefore, generalizations to other settings and other countries are limited. Finally, while the calculations of the relationships between clinical variables and sleep quality were adjusted for age and sex, further adjustment for other clinical variables could lead to even deeper insights into the relationships between clinical factors and sleep quality.

Given these limitations of the study, the following areas are proposed for further research: conducting longitudinal studies to examine the suitability of sleep items for measuring dynamic changes in sleep quality; conducting such studies in other cultural contexts to assess cross-cultural generalizability; and conducting studies to compare the results of such subjective single-item measures with those of objective sleep indicators.

In summary, the single-item assessments provided consistent and trustworthy results. Even if the reliability is lower than that of multi-item scales, individual sleep items with four response options can still provide useful and consistent information on sleep disturbances experienced by cancer patients. Such assessments can be used for detecting subgroups of vulnerable patients eligible for sleep interventions as well as identifying sociodemographic and clinical factors that are relevant for understanding sleep quality.

## Data Availability

The raw data supporting the conclusions of this article will be made available by the authors, without undue reservation.
